# Methyl jasmonate effectively enhanced some defense enzymes activity and Total Antioxidant content in harvested “Sabrosa” strawberry fruit

**DOI:** 10.1002/fsn3.300

**Published:** 2015-10-26

**Authors:** Mohammadreza Asghari, Ali Rashid Hasanlooe

**Affiliations:** ^1^ Department of HorticultureFaculty of AgricultureUrmia UniversityUrmiaIran

**Keywords:** Catalase, methyl jasmonate, peroxidase, polyphenol oxidase, strawberry, total antioxidant content

## Abstract

The use of chemicals in postharvest technology of horticultural crops is highly restricted and it is necessary to introduce safe food preserving methods. Strawberry is very susceptible to postharvest losses and more than 50% of harvested fruit is lost in Iran. Effect of postharvest treatment with methyl jasmonate (at 0, 8, and 16 *μ*mol L^−1^) on some quality attributes of Sabrosa strawberry fruit during storage at 1 ± 0.5°C with 90–95% RH for 14 days followed by 24 h at 20°C was studied. Methyl jasmonate, at both concentrations, decreased weight loss and retained marketability of fruits. Catalase activity of treated fruits was decreased during the first days, but showed a substantial increase during the second week. Methyl jasmonate, in a concentration‐dependent manner, enhanced peroxidase activity. Fruit total antioxidant capacity was enhanced by methyl jasmonate treatment. The results indicated that methyl jasmonate plays a key role in establishing resistance against stresses, enhancing fruit defense systems, antioxidant capacity, and storage life leading to decreased postharvest losses. This phytochemical has a good potential to be used in postharvest technology of Sabrosa strawberry fruit and enhance the fruit postharvest life.

## Introduction

Active oxygen species (AOS) and free radicals are the major players in aging and diseases like inflammation, arthritis, immune system impairment, different cancers, and heart disease, and much more focus has been given to the involvement of antioxidants in free radical scavenging and related senescence, and diseases prevention (Cai et al. 2004; Kaefer and Milner 2008; Huang et al. 2010). Similar to human cells, the aging and senescence process in plant cells is highly related to free radicals and AOS, and several antioxidants are responsible for detoxifying the plant cells from these dangerous agents. The antioxidant systems in plants and harvested crops include antioxidant enzymes such as superoxide dismutase (SOD, EC 1.15.1.1), ascorbate peroxidase (APX, EC 1.11.1.11), peroxidase (POD, EC 1.11.1.7), catalase (CAT, EC 1.11.1.6)) as well as nonenzymatic compounds with antioxidant activity such as ascorbic acid, glutathione, phenolic compounds, flavonoids, catkin*,* carotenoid, and *α*‐tocopherols (Asghari and Soleimani Aghdam 2010). Since natural antioxidants are more readily acceptable than the synthetic ones, fruits containing high antioxidants are of more commercial importance. Strawberry fruit is a rich source of natural antioxidants and phytochemicals, particularly anthocyanins, flavonoids, phenolic acids, and ellagic acid, which have potent antioxidant and anti‐inflammatory functions and also essential minerals making it as one of the most commercial horticultural crops (Rice‐Evans and Miller 1996; Heinonen et al. 1998). Fresh juice of strawberries has high oxygen radical absorbance activity against peroxyl radicals (ROO*), superoxide radicals (O*_2_^−^), hydrogen peroxide (H_2_O_2_), hydroxyl radicals (OH*), and singlet oxygen (*O_2_), and it is well demonstrated that the antioxidant activity is different among varieties (Wang and Jiao 2000).

With progress in aging and senescence of harvested crops, they become more susceptible to postharvest losses. Because of the consumption during free radical scavenging process, the antioxidant content of the harvested crops is decreased during postharvest stages leading to accelerated senescence. Strawberries are perishable fruits and more than 50% of produced strawberries are lost in Iran during postharvest handling, and because of using improper postharvest technologies, the remaining part received by the consumers has a very low nutritional quality. Then, the use of proper postharvest technologies is essential to decrease the adverse effects of stress conditions, rate of aging process, keeping nutritional quality, and enhancing storage life of strawberries.

Recently, because of the food safety issues and environmental concerns, use of synthetic chemicals in postharvest technology of horticultural crops is highly restricted and much more researches have been focused on generally regarded as safe compounds (GRASC). Jasmonic acid (JA) and it's methyl ester (methyl jasmonate (MeJA)) are important natural GRAS compounds playing key roles in several plant cell communication and signaling processes including ethylene production, defense responses against different biotic and abiotic stresses, anthocyanin, and other phytochemicals synthesis (Demole et al. 1962; Creelman and Mullet 1995). MeJA treatments have been shown to decrease the changes in physical attributes such as color, weight, firmness, and the amount of bioactive compounds (phenolic content, antioxidants), and enhance storage life in some fruits (Karaman et al. 2012; Concha et al. 2013). Strawberry fruits treated with MeJA has been reported to have higher total phenolics, anthocyanins, and total antioxidant capacity (TAC) after 12 days of cold storage (Ayala‐Zavala et al. 2005). Exogenous MeJA has been shown to enhance SOD, CAT, and APX activity in harvested loquat fruits leading to reduced $O_{2}^{-}$ and H_2_O_2_ content (Cao et al. 2009). Furthermore, increase in SOD activity in peaches and CAT activity in tomatoes has been reported as the result of postharvest MeJA treatment (Ding et al. 2002; Jin et al. 2009).

Enzymatic browning mainly caused by polyphenol oxidase (PPO, EC 1.10.3.2) is a main cause of postharvest losses in different fruits as well as strawberries. As a result of accelerated senescence and damaged cell membranes, PPO oxidizes the phenolics to dark brown colored polyphenols leading to a substantial decrease in overall quality and marketability (Gao et al. 2013; Zhou et al. 2015). Different biotic and abiotic stresses such as pathogens and chilling injury are responsible for oxidative burst in the cells of harvested fruits leading to increased membrane damage and subsequent PPO activity. Exogenous MeJA have been reported to activate some heat shock proteins (HSPs), which are preservative agents of plant cells against different stresses (Soleimani Aghdama et al. 2013). Decreased susceptibility to chilling injury and prevention of PPO activity has been reported as the result of treatment with MeJA in peach Fruit (Jin et al. 2009).

The effect of MeJA on antioxidant content and some physiological traits in some crops has been studied, but the exact mechanism of the effects has not been well demonstrated. The purpose of this study was to examine the effect of postharvest treatment with different concentrations of MeJA on TAC and some defense and antioxidative enzymes, and also postharvest life of “Sabrosa” strawberry fruit during cold storage to see if whole antioxidative system is affected or not, and also to determine the response of fruit during short‐ and long‐term storage.

## Materials and Methods

### Sample preparation

“Sabrosa” strawberry fruits (*Fragaria × ananassa* Duch. cv. Sabrosa) were harvested at commercial maturity from a commercial production greenhouse in Urmia (Iran) and transported to postharvest laboratory at Urmia University. Fruit were selected for uniformity of color and size, and any fruit with apparent injuries, disease, or infections were removed.

### Treatments with MeJA

MeJA was purchased from Sigma Co. (Sigma Aldrich, Germany). Treatments were performed at 20°C in five replicates by placing the strawberry in a 120 L container, in which the appropriated volume of MeJA to reach the desired concentration (8 and/or 16 *μ*mol L^−1^) was deposited on filter paper at the bottom of the container and then immediately hermetically sealed. Duration of the treatment was 16 h, after which the fruits from each replicate were randomized and sorted into five fruit lots. Control fruit received no treatment. Both control and treated fruits were put in 100 mL plastic jars and stored at 1 ± 0.5°C with 90–95% RH. for 14 days followed by 24 h at 20°C. Fruit quality attributes were measured after 7 days of cold storage plus 24 h at 20°C and the end of storage.

### Weight loss

Fruit weight was recorded several times during the storage period and expressed as percentage of water loss in comparison to initial weight.

### Determination of fruit firmness

Universal testing machine (TA.XT Plus; Texture Analyzer, Stable Micro Systems, UK) equipped with a 6 mmol cylinder probe (P/6) and a 5 kg load cell was used to determine the fruit firmness. Test condition consisted of a 10 mm probe displacement distance (constant strain), 2 mm sec^−1^ pretest speed, 1 mm sec^−1^ test speed, and 10 mm sec^−1^ posttest speed. Penetration force was calculated in each case on the force–time curve.

### Marketability (overall quality)

Overall quality (percentage of fruit surface area decayed, shrunken and adversely affected) was considered as fruit marketability index and evaluated by 10 trained panelists using a 1–5 scale, where 1 =  unacceptable (>50% surface affected), 2 =  bad (20–50% surface affected), 3 =  acceptable (5–20% surface affected), 4 =  good (up to 5% surface affected), and 5 =  excellent (no decay, shrinkage, or any other adverse effects on fruit surface were seen). Results were expressed as an overall quality index.

### Determination of total antioxidant content (TAC)

Fruit juice TAC was determined by ferric ions reducing antioxidant power assay (FRAP) according to Benzie and Strain (1996) with slight modifications. The stock solutions included 5 mL of a 10 mmol L^−1^ TPTZ (2, 4,6‐ tripyridyl‐ s‐ triazine) with 40 mmol L^−1^ HCL plus 5.41 mL of FeCl_3_ (20 mmol L^−1^) and 50 mL of phosphate buffer, (0.3 mol L^−1^, pH = 3.6), and was prepared freshly and warmed at 37°C. Fruit extracts (150 mL) were allowed to react with 2.85 mL FRAP solution and the absorbance of reaction mixture at 593 nm was measured spectrophotometrically after incubation at 37°C for 10 min. For construction of calibration curve, five concentrations of FeSO_4_7H_2_O (1000, 750, 500, 250, 125 *μ*mol L^−1^) were used to obtain the calibration curves. The values were expressed as the concentration of antioxidants having a ferric reducing ability equivalent to that of 1 mmol L^−1^ FeSO_4_. (*y* = 0.0009*x* − 0.0275, *R* ^2^ = 0.995).

### Determination of CAT enzyme activity

All enzyme extract procedures were conducted at 4°C. CAT activity was measured according to the Beers and Sizer (1952) with slight modifications. The reaction mixture consisted of 2.5 mL sodium phosphate buffer (50 mmol L^−1^, pH 7.0), 0.2 mL H_2_O_2_ (1%), and 0.3 mL enzyme. The decomposition of H_2_O_2_ was measured by the decline in absorbance at 240 nm. The specific activity was expressed as U mg^−1^ protein, where one unit of catalase converts one mol of H_2_O_2_ per min.

### Determination of POD enzyme activity

In order to determine the POD activity, the reaction mixture contained 2.5 mL of 50 mmol L^−1^ phosphate buffer (PH 6.1), 1 mL of 1% hydrogen peroxide, 1 mL of 1% guaiacol, and the enzyme extract. The increase in absorbance at 490 nm was followed up for 1 min (Updhayaya et al. 1985).

### Determination of PPO enzyme activity

The PPO activity was assayed by determining the rate of increase in absorbance at 420 nm and 25°C. The reaction mixture contained 0.5 mL of enzyme extract to 2.5 mL of buffered substrate (100 m mol L^−1^ sodium phosphate, pH 6.4, and 50 mmol L^−1^ Catechol). The linear section of the activity curve as a function of time was used to determine the PPO activity (U mg^−1^ protein min^−1^). The unit for the PPO activity was defined as a change of 0.001 in absorbance at the conditions of the assay (Pizzocaro et al. 1993).

### Statistical analysis

The experiment was designed as a completely randomized design with 3 (MeJA concentration) × 2 (storage time) = 6 factors with 5 replicates (each replication was included 10 fruits). ANOVA was performed for the experiment using SAS. Means of data were compared by Duncan's Multiple Range Test. Differences at *P* ≤ 0.05 were considered significant. A summary of the statistical results is shown in Table 1.

**Table 1:** Effect of postharvest MeJA treatment on weight loss, firmness, and marketability index of “Sabrosa” strawberry fruit

Treatments		Quality attributes		
MeJA (*μ*mol L^−1^)	Storage time (days)	Weight loss (%)	Firmness (N cm^−2^)	Marketability
0	–	0.87^a^	0.41^a^	2.82^b^
8	–	0.65^b^	0.36^b^	3.52^a^
16	–	0.67^b^	0.32^b^	3.42^a^
		**	**	**
–	8	0.51^b^	0.41^a^	3.75^a^
–	15	0.95^a^	0.31^b^	2.75^b^
		**	**	**
0	8	0.59^a^	0.49^a^	3.42^a^
0	15	1.16^a^	0.32^c^	2.22^a^
8	8	0.42^a^	0.39^b^	3.96^a^
8	15	0.89^a^	0.32^c^	3.08^a^
16	8	0.53^a^	0.34^bc^	3.88^a^
16	15	0.81^a^	0.29^c^	2.96^a^
		ns	*	ns

Means followed by different letters within a group are significantly different at 5% level.

** and * represent significance at the 0.01, and 0.05 levels, respectively, and NS represents nonsignificance at *P* < 0.05.

## Results

### Weight loss

As shown in Table 1, the weight of control fruit was rapidly decreased during storage, but MeJA, at both concentrations, effectively decreased the rate of weight loss. There was no significant difference between the two levels of MeJA in retaining fruit weight.

### Firmness

After 14 days of storage at 1 ± 0.5°C followed by 24 h at 20°C, the firmness was decreased in all fruits, but the control fruits showed a substantially increased firmness value due to water loss and lignification.

### Fruit marketability index

Since overall quality is the most important factor determining the marketability, it was considered as the marketability index. As shown in Table 1, fruit treated with MeJA showed higher marketability than control fruits and there was no significant difference between the two levels of MeJA in retaining fruit marketability index.

### TAC

Fruit TAC was significantly affected by exogenous MeJA treatment. As shown in Table 2, fruit treated with 8 *μ*mol L^−1^ MeJA had the highest TAC after storage for 8 and 15 days. The difference between the two MeJA levels was significant after 8 days, but there was no significant difference between them after 15 days.

**Table 2:** Effect of postharvest MeJA treatment on TAC and CAT, POD and PPO enzymes activity of “Sabrosa” strawberry fruit

Treatments		Quality attributes			
MeJA (*μ*mol L^−1^)	Storage time (days)	TAC (mmol Fe^+2^ 100 g^−1^ FW)	CAT (U mg^−1^ protein)	POD (U mg^−1^ protein)	PPO (U mg^−1^ protein)
0	–	379.33^b^	122.805^a^	165.90^b^	7791.20^b^
8	–	579.78^a^	115.872^a^	249.62^a^	10132.00^a^
16	–	385.22^b^	114.046^a^	269.53^a^	8464.00^b^
		**	ns	**	**
–	8	498.85^a^	104.93^b^	243.61^a^	9050.10^a^
–	15	397.37^b^	130.20^a^	213.09^a^	8541.30^a^
		**	**	ns	ns
0	8	406.78^bc^	118.25^b^	168.66^a^	7374.40^b^
0	15	351.89^c^	127.35^ab^	163.12^a^	8208.00^b^
8	8	695.22^a^	96.33^c^	263.77^a^	11464.00^a^
8	15	464.33^b^	135.40^a^	235.47^a^	8800.00^b^
16	8	394.56^bc^	100.22^c^	298.39^a^	8312.00^b^
16	15	375.89^bc^	127.86^ab^	240.66^a^	8616.00^b^
		**	**	ns	*

Means followed by different letters within a group are significantly different at 5% level.

** and * represent significance at the 0.01, and 0.05 levels, respectively, and NS represents nonsignificance at *P* < 0.05.

### CAT enzyme activity

During the first week the activity of CAT enzyme, as an important antioxidant and also the key enzyme in systemic resistance network, was decreased in treated fruits, but it was increased in all fruits during the second week (Table 2). Although MeJA at both concentrations was effective in enhancing CAT activity, it was more significantly enhanced by 8 *μ*mol L^−1^ MeJA.

### POD enzyme activity

**Figure 1:**
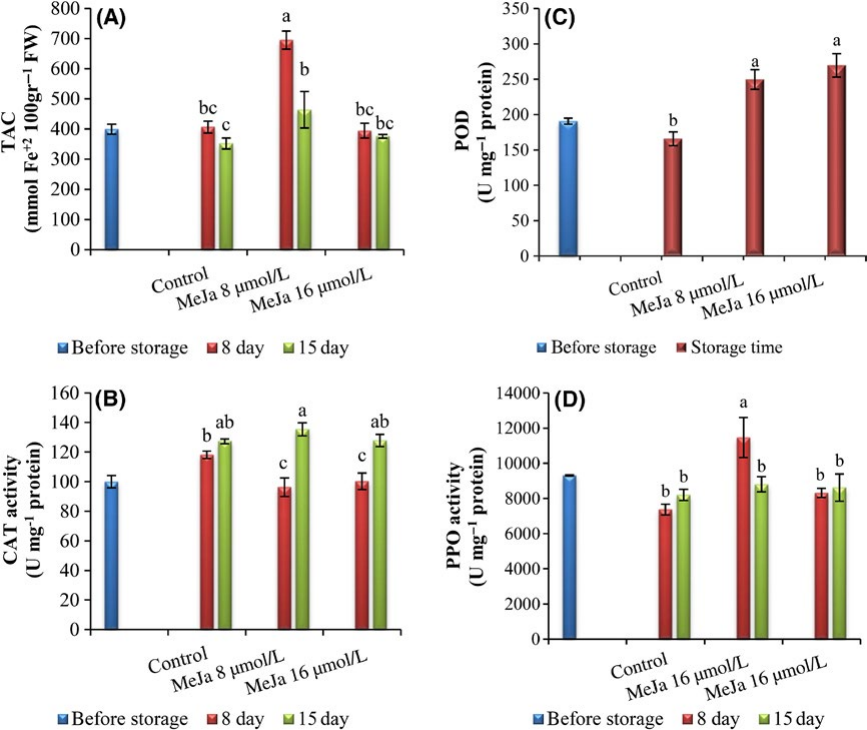
Effects of postharvest MeJA treatment on total antioxidant capacity (A), catalase (B), peroxidase (C), and polyphenol oxidase (D) enzymes activity of “Sabrosa” strawberry fruit after 8 and 15 days of cold storage followed by 1 day at 20°C. Means followed by different letters are significantly different at 5% level.

As shown in Table 2 and Figure 1, control fruit showed a decrease in POD activity but MeJA, in a concentration‐dependent manner, effectively enhanced the enzyme activity during the storage period. The activity of POD in both treated and control fruit was significantly decreased during the second week of cold storage.

### PPO enzyme activity

After 14 days of storage at 1 ± 0.5°C followed by 24 h at 20°C, PPO enzyme activity declined in all fruits. According to Figure 1, at the end of first week, fruit treated with 8 *μ*mol L^−1^ MeJA showed an increase in PPO enzyme activity, but at the end of storage, there was no significant difference between the treated and control fruit.

## Discussion

Fresh fruit and vegetables are living tissues which continue to lose weight due to water loss and respiration after harvest, which is a serious problem in harvested crops (Hung et al. 2011). Fruit flesh firmness is one of the most important indicators for shelf life, preservation potential, consumer acceptance, and market value of horticultural crops (Valero et al. 2003). Ethylene is known as the ripening hormone promoting pectin degradation leading to crop flesh softening together with increase in the rate of various other ripening processes such as discoloration, weight loss, general senescence, and respiration (Singh and Khan 2010). There are conflicting data in different literature about the effects of jasmonates on ethylene production and action in different horticultural crops. It seems that jasmonates may enhance ethylene production in some fruits while blocking its effects. Under different stress conditions, including postharvest storage and transport situations, plant and harvested crop cells may produce jasmonates via membrane lipids breakage. In fact, under these conditions, plant cells undergo jasmonates production as an important defense mechanism. While exogenous MeJA treatment my act as a downregulating factor for internal jasmonates production leading to decreased internal jasmonates production and membrane lipid preservation. As an important result, the retention of membrane lipids is the major reason for blocking ethylene effects, preventing water loss, and decreasing respiration and other senescence‐related processes rates such as softening (Creelman and Mullet 1995).

Antioxidant content is a good indicator of internal cell situation. Fruit with high antioxidant content are in a good condition of health and are considered as marketable. Free radicals and AOS are produced during normal cell metabolism and also in response to biotic and abiotic stresses, and antioxidants are consumed for scavenging free radicals and AOS. With progress in senescence and increase in metabolic activities, the rate of free radicals and AOS production is increased. In comparison to whole plant systems, the capacity of harvested fruit to produce antioxidants is limited, and with progress in ripening and senescence, the rate of free radical and AOS production precedes the antioxidant systems leading to cell damage and subsequent postharvest losses. Then any factor decreasing cell metabolic activities and contributing in defense systems against different stresses will decrease the rate of free radical production and senescence leading to antioxidant preservation. It has been demonstrated that exogenous treatment with MeJA enhances the antioxidant capacity of different harvested fruits and horticultural crops (Wang and Zheng 2005; Chanjirakul et al. 2006; Wang et al. 2008; Sayyari et al. 2011). In this study, MeJA not only enhanced strawberry fruit TAC, but also increased the activity of CAT and POD as two important antioxidant and defense enzymes. Similar results were also observed in peaches (Yao and Tian 2005; Jin et al. 2009), cherrie (Yao and Tian 2005) and loquat fruits (Cao et al. 2008). It is well‐known that during the local and systemic acquired resistance in disease and stress‐resistant crops, CAT activity decreases in first steps allowing the H_2_O_2_ to increase as a second messenger, and also as the major player in hypersensitive response, and after the establishment of a resistance system, increase in the activity of CAT is necessary to eliminate the elevated dangerous H_2_O_2_ molecules (Asghari and Soleimani Aghdam 2010). It has been proposed that JA may play a direct or indirect role in systemic resistance network. Also it is well‐known that JA activates phenylalanine amonia‐lyase (PAL, EC 4.3.1.24) as the key enzyme in phenolics and salicylic acid biosynthesis, the later considered as the main activator of local and systemic acquired resistance in different plants. Our data indicates that MeJA directly affects the systemic resistance due to the decreasing CAT activity. According to our findings, postharvest MeJA treatment decreased CAT enzyme activity during the first 8 days of storage but enhanced it later, coinciding with the mentioned acquired resistance pattern. POD and PPO are enzymes with important roles in enhancing antioxidant capacity and triggering some resistance systems against biotic and abiotic stresses in plants and harvested crops. Both catalyzing the formation of different antimicrobial phenolic substances such as quinones which are toxic to pathogens (Mohammadi and Kazemi 2002). In addition, POD and PPO activities have been considered as important biochemical markers for crop resistance against different stresses specially the pathogens (Mohammadi and Kazemi 2002). Significant increase in PPO activity also may negatively affect the color of strawberries due to formation of dark brown pigments. According to our data, after an increase during the first week of storage, PPO activity was decreased in the second week indicating that MeJA treatment will not result in a loss in fruit color during long‐term storage.

The results of this study indicated that MeJA enhances fruit postharvest life due to enhanced total antioxidant capacity, decreased weight loss, and senescence rate. It is proposed that MeJA may effectively contribute in systemic acquired resistance establishment via decreasing CAT activity during first week and then increasing it during the second week. In addition, the useful effects of MeJA in enhancing fruit postharvest life and decreasing senescence rate by phenolics, and some HSPs, as main cell protecting agents, should be considered.

## Conclusion

The results of this study indicate that MeJA directly plays key roles in enhancing fruit storage life, antioxidant and defense systems, and treatment of harvested fruits with MeJA, as a safe phytochemical, may significantly enhance “Sabrosa” strawberry fruit storage life, quality attributes, and nutritional quality by enhancing the fruit's whole antioxidant capacity and defense enzymes activity. The suitable concentration of MeJA for enhancing TAC and antioxidant enzymes activity in Sabrosa strawberry was 8 *μ*mol L^−1^.

## Conflict of Interest

None declared.
